# Two Types of Medical Leader

**Published:** 1917-11-10

**Authors:** 


### TWO TYPES OF MEDICAL LEADER.

### No. 2.—The Humbug.

By CADUCEUS.

"TALENT? Merit? Services?" we read in "Télémaque"), "Bah, belong to a coterie!" Some human beings know all about this maxim of themselves, before they are old enough to read anything.

Years ago, Tom Compton was the shrewd, selfish, thick-skinned, old-young son of a successful provincial timber merchant who did a little joinery, and even, in early days, a little undertaking. And as Tom preferred the parlour to getting sawdust on his clothes in the office, and could never be shamed into foregoing any of his tastes, the understanding somehow arose and strengthened that he was entitled to a start in a profession. At first there was uncertainty as to which profession should be chosen. The Church was not discussed much, because, although smooth-spoken enough, the lad was plainly too selfish and cared nothing for books. The Army was rather too aristocratic and so was the Bar; but, as Whistler said of a certain popular painter, Tom "must have tossed up" when he missed being an attorney. He did miss; and only medicine was left. Tom's father, following a prosperous tradesman's instinct to do things properly, sent him to Oxbridge, a proceeding a good deal rarer then than now. At Oxbridge the trustworthy son took a poll degree, and one of those small distinctions always securable by means of moderate application backed by tact and self-assertion cunningly blended. He walked a London hospital a little, passed for a regulation diploma, and came straight back to his native town. For there beyond a doubt it was that his opportunity awaited him; besides which, both London and the University gave every sign of preferring others.

The honorary appointment at the local hospital, to get which was child's play to such a man, did not at that time and place mean much of itself, at all events for a good few years. But in the accompanying potentialities lay the making of his career. A decade before, a new University had been founded, and most doctors on the staff of this hospital then became *ipso facto* teachers to a faculty recognised by the authorities governing British medical education. The town had been producing in every generation for time out of mind tinkers and tailors, butchers and bakers, merchants and traders: now all of a sudden it produced a crop of professors. But the real evil was not this mushroom character—the faculty might even have been the better for that—but the presence of certain indications of the toadstool. Academic recognition and honours and representation indeed flowed to or opened out in sure prospect before this narrow circle of sons of leading townsmen, which latter were finding a good deal of the money, and therefore, in a manner of speaking, having a good deal of say in calling the tune. As for Thomas, he found it good, served on every committee possible, crept up and up, and in and in; while colleagues who had made a professional name for themselves mostly went on elsewhere, and brilliant young students with no backing sank into club practice.

Medicine proper is not the test of performance that surgery is, and so before very long, being, as he remained, a bachelor—the proposal for an advantageous marriage was refused by the lady—he was putting by the two or three hundreds a year that enable a man to make himself a well-placed pensioner of the community in later life. A fair good clinician is their description of him at that time; not at all incompetent, but with small right to wag his head in a pulpit. He produced a pamphlet or two which show the grade of work which would be lost by cutting down external incentives; but, as his nature (with its basic affinity to the world's slow stain) was, he kept himself in evidence much more by putting in appearances than by committing himself on paper or by trying to do something. Punctual, businesslike, an excellent secretary, it was only in the French sense of the word that he assisted at committee work, however. Whoever wanted to advance matters had to push Tom's deadweight; at most he got out and walked, when he saw the majority determined to take risks to get progress. But when the new laboratory, or whatever it was, was actually an accomplished thing, then who but he for pulling out scarf and hood and gown and playing a leading and fervent part in the inaugural ceremony, especially in the group photograph? His voice inflection early became as artificial as a butler's, a habit which played him false, for in old age it got to be ludicrous, echo-provoking; and, what also kept equals at a safe distance, he was delivered of outrageous platitudes quite unashamedly and automatically. How thoroughly his disposition delighted in that regrettable phenomenon, the intrinsic insincerity of conversation! How seldom he contributed to a discussion any remark with the slightest life or risk about it! How frequent the lies he told with his manner, safe way of humbugging interlocutors! But, as is common with biographers, I am catching my subject's style. After forty years of all such tricks, never having it remembered of him that he gave himself away (which unfortunately breeds prestige), Royalty came down to open the new hospital, and Tom was knighted, just as the Hero. And, after all, at an earlier age than Francis Galton.

Now he was a pseudo-celebrity. Although he couldn't have told you Savigny's name from Adam's, he was nevertheless a full-blown Vice-Chancellor, standing beside famous men when they did the young University a good turn by accepting its honorary degrees. He used to go up to London, too, and, not to betray himself or darken counsel, vote with the majority on important issues

☛ The first article appeared in "The Hospital" of October 6, 1917.

**TWO TYPES OF MEDICAL LEADER**—(*continued*).

of which he knew nothing. He mouthed out lectures, years out of date and transcribed from an easily accessible text-book at that, through which the students with youthful mercilessness would snore or lark, and which, a keen junior colleague blurted out in conversational hyperbole, were the biggest scandal in that part of England. But the mischief did not end with personal shortcomings. True, all his life he borrowed prestige from position and never paid back, much less contributed, any. But what was more lastingly disastrous to the fame of this seat of learning was this its professor's example and influence and cast of vote. His methods are not hard to copy. As schools of philosophy commonly split up in time into two wings, so you could see one disciple strutting and bluffing and pouring out a cuttle-fish flood of unexceptionable propositions, such as that our knowledge of syphilis had been profoundly modified by the discovery of the spirochæte; and another a sheer wirepuller, who, observing a colleague keeping private notes of cases, asked him in all seriousness why he did so. Such men took, so to say, the extinguisher from Thomas' failing hand.

Of course, public opinion was not blind. Our friend—another of his means of self-assertion—had an iconography in stone, in bronze, in wood, in paint, in hundreds of photographs; and in many of these media was expressed the spirit of the general judgment. It is a glorious function of the artist to put his tongue in his cheek, to avenge poor smothered truth. And at academic dinners there would levitate, to repose squalidly on the head of the bust, a bit of orange-peel. But when people ascribe the eminence of Thomas to the easier conditions prevailing formerly, to the good hands one used to get then, I always differed. It is true, of course, that he had nothing to do with the success of the institution. That was due firstly to the rise of modern surgery; secondly to the preference of the public for any sort of degree over a diploma, and for keeping medical student sons at home under their eye, and where they could push them, over letting them loose in London to face the keenest competition; and thirdly to a surgeon of real competence. But all the same, Thomas was (happily) exceptional. First of all, as he himself would have reflected when joyless faces met his wreathed smiles, his father was better off than most doctors' fathers. Then his character was strong. You could see it in the big cheek-bones—a never-failing sign, as Dumas says, of audacity and craftiness. I have noticed their like in a billiard champion, a little in Cagliostro's portraits, but most of all in those illustrations to "Huckleberry Finn" portraying the old rascal who called himself "the King." Dumas, by the way, gives them to a fraudulent banker. A friend of mine once described Thomas as a mixture of Aristotle's Magnificent Man and Sir Pertinax MacSycophant; but for the first ingredient I myself would substitute Charles Lamb's vestryman uncle, he whom a pompous manner and two mispronounced Latin words raised to "the highest parochial honours St. Andrews has to bestow." The second one there is no doubt about. Ah, Sir Pertinax, when will the world learn to rid itself of you? And, what is more pressing, what makes the Humbug a good subject in these days of reconstruction, when will England—before her reputation for achievement falls ruinously low?

